# A Randomized Clinical Trial of ICT-based Interventions for Sodium and Potassium Regulation in Healthy Adults

**DOI:** 10.1093/ajh/hpaf049

**Published:** 2025-04-11

**Authors:** Yuichiro Yano, Kaori Kitaoka, Takayoshi Ohkubo, Tomonori Okamura, Hiroshi Kanegae, Katsushi Yoshita, Rumi Tsukinoki, Yukiko Okami, Koichi Node, Hiromi Rakugi, Hiroshi Itoh, Katsuyuki Miura

**Affiliations:** Department of Advanced Epidemiology, NCD Epidemiology Research Center, Shiga University of Medical Science, Otsu, Japan; Department of General Medicine, Juntendo University Faculty of Medicine, Tokyo, Japan; Artificial Intelligence Incubation Farm (aif), Juntendo University Faculty of Medicine, Tokyo, Japan; Department of Family Medicine and Community Health, Duke University, Durham, NC, USA; Department of Advanced Epidemiology, NCD Epidemiology Research Center, Shiga University of Medical Science, Otsu, Japan; Department of Hygiene and Public Health, Teikyo University School of Medicine, Tokyo, Japan; Department of Preventive Medicine and Public Health, Keio University School of Medicine, Tokyo, Japan; Genki Plaza Medical Center for Health Care, Tokyo, Japan; Department of Nutrition, Graduate School of Human Life and Ecology, Osaka Metropolitan University, Osaka, Japan; Department of Public Health Nursing, Institute of Science Tokyo, Tokyo, Japan; Department of Preventive Medicine, NCD Epidemiology Research Center, Shiga University of Medical Science, Otsu, Japan; Center for Food Science and Wellness, Gunma University, Maebashi, Japan; Department of Cardiovascular Medicine, Saga University, Saga, Japan; Japanese Society of Hypertension, Tokyo, Japan; Japanese Society of Hypertension, Tokyo, Japan; Japan Organization of Occupational Health and Safety Osaka Rosai Hospital, Sakai, Japan; Japanese Society of Hypertension, Tokyo, Japan; The Center for Preventive Medicine, Keio University, Tokyo, Japan; Department of Preventive Medicine, NCD Epidemiology Research Center, Shiga University of Medical Science, Otsu, Japan; Japanese Society of Hypertension, Tokyo, Japan

**Keywords:** blood pressure, ICT-based interventions, self-monitoring, spot urine sodium-to-potassium ratios

## Abstract

**BACKGROUND:**

There is limited knowledge regarding effective strategies, including information and communication technology (ICT)-based interventions, to reduce sodium intake and increase potassium intake in healthy individuals.

**METHODS:**

We conducted a 3-month randomized controlled trial involving healthy adult employees with spot urine sodium-to-potassium ratios (spot UNa/UK) ≥4.0 or estimated 24-hour salt intake ≥10g. Estimated 24-hour UNa and UK were calculated using the Tanaka formula. Participants were assigned to one of four groups: (i) online education, where participants monitored their spot UNa/UK and received feedback from dieticians (*n* = 84); (ii) messaging, with similar self-monitoring and dietician messages (*n* = 84); (iii) self-learning, provided with an educational leaflet (*n* = 87); and (iv) a control group (*n* = 87). The primary outcome was the change in spot UNa/UK ratios, and secondary outcomes included changes in estimated 24-hour UNa and UK. The trial protocol specified a hierarchical order for testing the interventions, anticipating the highest efficacy in the online education group.

**RESULTS:**

After the intervention, the online education group showed a decrease in spot UNa/UK ratios (mean −0.9 (95% CI: −1.8 to 0.0), *P* = 0.052) compared to the control group. The increase in estimated 24-hour UK excretion was larger in online education compared to the control group (mean + 2.5 mmol/day (95% CI: −0.3 to 5.3), *P* = 0.085). The difference in estimated 24-hour UNa excretion between the online education and control groups was −4.3 mmol/day (95% CI: −15.5 to 6.9, *P* = 0.45).

**CONCLUSIONS:**

Combining self-monitoring of sodium and potassium intake with ICT-based interventions, including online nutritional education, was associated with a modest reduction in the estimated ratios of sodium and potassium intake in healthy individuals.

**CLINICAL TRIAL REGISTRATION:**

Japan Registry of Clinical Trials; 1032210217, https://jrct.niph.go.jp/en-latest-detail/jRCT1032210217

## Graphical Abstract

**Graphical Abstract:**
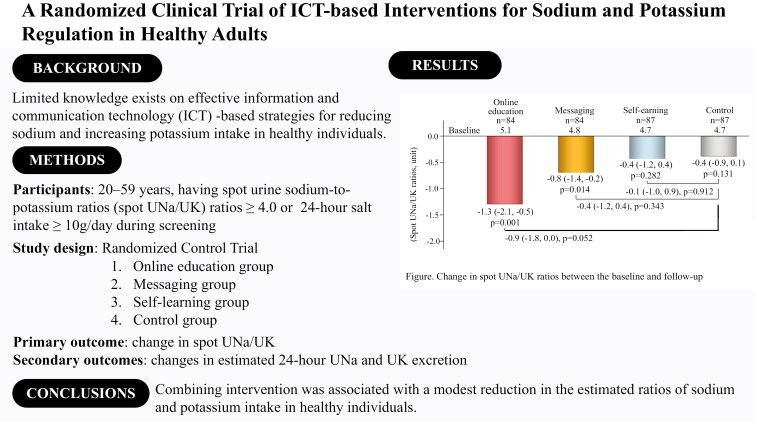

Excess dietary sodium and lower potassium intake are related to several chronic diseases, including hypertension and cardiovascular diseases (CVDs).^1,2^ Randomized trials of dietary sodium reduction and dietary potassium supplementation have shown an association with lowering blood pressure (BP) and decreasing CVD.^3–5^ Most guidelines and health organizations suggest a sodium intake, defined as <2–2.4 g of sodium (equivalent to <5–6 g of sodium chloride) per day, to reduce high BP and improve cardiovascular outcomes.^6,7^ However, poor adherence to recommendations for dietary sodium reduction has been reported worldwide.^8^

Estimates of sodium and potassium intake using spot urine (spot UNa and UK) have been linked to BP,^9^ and the sodium-to-potassium ratios (UNa/UK ratios) are more strongly associated with BP compared to estimates of UNa or UK alone.^9–12^ Furthermore, recent longitudinal studies demonstrated that a reduction in UNa/UK ratios in spot urine was associated with a reduction in BP.^12,13^ However, it is not known whether (i) strategies for involving healthy individuals in self-monitoring their sodium and potassium intake will lead to reduced UNa/UK ratios.

Digital tools, including information and communication technologies (ICTs), may offer promising strategies to improve adherence to dietary recommendations by providing personalized feedback and interactive communication. However, their efficacy in facilitating dietary sodium reduction and potassium increase has not been fully explored.

We conducted this randomized controlled trial (RCT) with healthy adults with high sodium or low potassium intake to evaluate three distinct intervention strategies: (i) online nutritional education, offering personalized feedback; (ii) text messaging, providing concise reminders to enhance adherence; and (iii) self-learning through educational leaflets, representing a traditional, noninteractive approach. These strategies were designed to assess the impact of varying levels of interactivity and engagement on dietary behavior changes.

## METHODS

**Figure 1.**
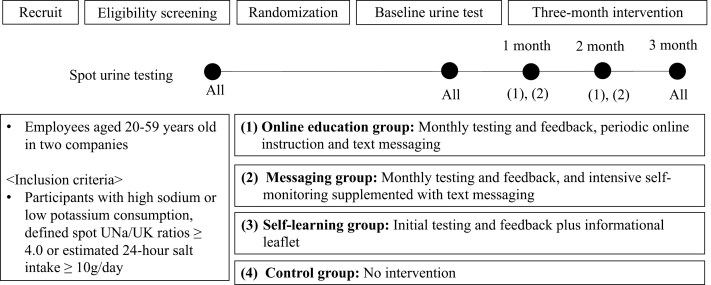
Study protocol. The study enrolled participants with high sodium consumption or low potassium consumption, defined by spot UNa/UK ratios ≥4.0 mmol/mmol or estimated 24-hour UNa ≥10 g/day in the screening urine test. Participants were assigned to one of the following groups for 3 months: (i) online education group, (ii) messaging group, (iii) self-learning group, and (iv) control group. Differences in the interventional components received by each group are described below and summarized. Randomization (1:1:1:1) and group assignment were performed automatically using a web-based block randomization and electronic data capture system. Randomization was stratified by the following factors: age (>40 years old), sex, and the proportion of those who had specific health guidance at their latest annual health checkup. Individuals were assigned randomly to groups, with notifications of group assignments being sent individually via email. UNa, urinary sodium; UK, urinary potassium.

Participants were enrolled in the current study from July 2021 through September 2021. The exclusion criteria included persons with chronic kidney disease (since limiting potassium intake is often recommended),^14^ those with a loss of appetite, and pregnant women. The current RCT (jRCT1032210217) recruited employees in two companies (i.e., **Daiwa Securities Group Inc.** and Japan Airlines Co., Ltd.). As shown in Figure 1, the study enrolled participants with high sodium consumption or low potassium consumption, defined by spot UNa/UK ratios ≥4.0 mmol/mmol or estimated 24-hour UNa ≥10 g/day in the screening urine test. Participants were assigned to one of the following groups for 3 months: (i) online education group, (ii) messaging group, (iii) self-learning group, and (iv) control group. Differences in the interventional components received by each group are described below and summarized briefly in Figure 1. Randomization (1:1:1:1) and group assignment were performed automatically using a web-based minimization method and electronic data capture system. Randomization was stratified by the following factors: age (>40 years old), sex, and the proportion of those who had specific health guidance at their latest annual health checkup. Individuals were assigned randomly to groups, with notifications of group assignments being sent individually via email. The trial was approved by the ethics committee of the Japanese Society of Hypertension and was conducted according to the principles of the Declaration of Helsinki and Good Clinical Practice guidelines. All the participants provided electronic informed consent before participating in any of the experiments.

### Intervention

Participant groups and interventional variables are provided in Supplementary Material. Briefly, participants were assigned to one of four groups. The Online Education Group received personalized dietary guidance via video or online sessions (baseline, Months 1, 2, and end), spot urine tests at Months 1 and 2 reviewed by dieticians, biweekly text messages (Supplementary Table S1), a dietary booklet, and set dietary goals (Supplementary Table S2) with incentives for self-reported achievement. The Messaging Group underwent spot urine tests at Months 1 and 2, received test results, set dietary goals, and received biweekly text messages, with incentives for self-reported achievement (Supplementary Table S3). The Self-Learning Group received a dietary leaflet and their baseline urine test results but had no further urine tests, online education, or text messages. The Control Group received no urine test results, dietary guidance, or study-related materials during the intervention.

### Estimation of 24-hour sodium and potassium intake

During both the baseline assessment and resurvey visit conducted 3 months after baseline, initial morning spot urine samples were collected from all participants (see Supplementary Figure S1 for urine collection procedures). Samples were transferred from the collection vessels to the coordinating center laboratory (Healthcare Systems Co., Ltd) on the day of sampling. Urinary analyses were performed within 72 hours after sample collection using a TBA120FR (CANON MEDICAL SYSTEMS CORPORATION). The concentration of urinary sodium and potassium was measured using the electrode method, and urinary creatinine (Cre) using an enzymatic method. The details of the estimation formulas for 24-hour UNa and UK excretion, as well as estimated salt and potassium intake calculations, are provided in Supplementary Material.

### Other measurements

The details of other measurements, including BP, are provided in Supplementary Material.

### Outcomes

The primary outcome was a change in spot UNa/UK ratios from randomization to Month 3. Secondary outcomes were changes in estimated 24-hour UNa and UK excretion.

### Statistical analysis

A prior study reported that strategies involving self-monitoring of sodium and potassium intake led to reduced UNa/UK ratios in spot urine by 1.0 (standard deviation (SD), 1.5) in healthy individuals. With a bilateral significance level of 1.7% and detection power of 90%, the required sample size was 62 participants per group. Assuming a 15% dropout rate, a total of 320 participants (80 participants per group) were required for the current trial.^15^

Three analysis sets were defined, including the intention-to-treat analysis (primary) and the full analysis set (secondary). Additional details are provided in Supplementary Material. The full analysis set excluded participants with missing baseline urine tests and was conducted on the remaining individuals. Assuming that all missing data for urinary measurements were assumed to be missing at random, we imputed missing data for urinary data at baseline and Month 3 (Supplementary Table S4), using a fully conditional specification method.^16^

To assess the effectiveness of the intervention, mixed models were used to compare the baseline and follow-up data within each group. Changes between the baseline and follow-up period in the intervention and control groups were measured using the mean and 95% confidence interval (CI) at baseline and Month 3. For adjustment of multiplicity, the hypotheses were tested according to a hierarchical strategy, where the online education group is of greatest importance, but convincing results in the messaging group and self-learning group would add to our understanding of the value of the various treatment components. The hierarchical order for testing null hypotheses was prespecified in the trial protocol. Since no reduction or splitting of α is necessary in a hierarchical strategy, statistical significance was defined as a *P* value >0.05 using two-sided tests using SAS version 9.4 software (SAS Institute, Cary, NC).

As per the preestablished statistical analysis plan, we tested for heterogeneity in the associations between each intervention group and outcomes by each subgroup categorized according to sex, age (<40 vs. ≥40 years), body mass index (<25 vs. ≥25 kg/m^2^), baseline UNa/UK ratios and estimated 24-hour UNa and UK excretion above or below median levels, and study location. These were conducted by incorporating multiplicative interaction terms. When a statistically significant interaction was observed (*P* value <0.05), stratified analyses were conducted.

We conducted three sensitivity analyses. First, we performed analyses without imputing missing urinary measures. Second, when baseline urinary measures were missing, we used urinary measures obtained at screening exams (Figure 1). Third, we adjusted for baseline urinary data, age, sex, and variables obtained in the health checkups (i.e., body mass index, eGFR, hypertension, and diabetes). These were selected a priori because they may be associated with sodium and potassium intake and excretion.^17–19^

## RESULTS

### Trial population

Of the 702 individuals who provided electronic informed consent and were screened for eligibility, we excluded 6 who were ineligible, 26 who were missing a screening urine test, and 328 with spot UNa/UK ratios <4.0 and estimated 24-hour salt intake <10 g/day. The final sample size for participants who underwent randomization was 342. The mean age of the study population was 42.0 years (SD, 11.0), 48.8% (167) were female, and 16.1% (55) had hypertension. Mean (SD) baseline levels of estimated 24-hour UNa and UK excretion were 149.5 (35.3) mmol/day and 33.7 (8.1) mmol/day, respectively. The mean (SD) baseline level of spot UNa/UK ratios was 4.8 (2.9). Distributions of spot UNa/UK ratios and estimated 24-hour UNa and UK excretion are shown in Supplementary Figure S2. The flow diagram illustrating the derivation of the sample for the analyses is shown in Supplementary Figure S3 in Supplemental Material. Of the 342 participants, 84 were randomized to the online education group, 84 to the messaging group, 87 to the self-learning group, and 87 to the control group. Demographic characteristics of the participants at randomization were similar across groups except for the prevalence of diabetes between the self-learning and control groups (Table 1).

**Table 1.** Characteristics of participants

	Online education *n* = 84	Messaging *n* = 84	Self-learning *n* = 87	Control *n* = 87
Characteristics obtained at baseline				
Age, years	41.8 ± 10.2	41.7 ± 10.8	42.4 ± 11.9	42.2 ± 11.2
Men, %	42 (50.0)	43 (51.2)	45 (51.7)	45 (51.7)
Urinary Na, mEq/L	120.0 ± 55.0	113.6 ± 56.0	116.6 ± 57.9	118.5 ± 58.0
Urinary K, mEq/L	30.2 ± 21.9	29.3 ± 19.9	30.6 ± 18.8	29.3 ± 16.3
Urinary Cr, mg/dL	122.5 ± 66.0	123.9 ± 70.2	127.7 ± 78.5	119.9 ± 59.9
Urinary Na/K ratio, mmol/mmol	5.1 ± 3.3	4.8 ± 2.5	4.7 ± 3.3	4.7 ± 2.3
Estimated 24-hour urinary Na excretion, mmol/day	150.4 ± 35.9	148.6 ± 34.4	148.1 ± 37.6	150.7 ± 33.8
Estimated 24-hour urinary K excretion, mmol/day	33.4 ± 8.4	33.2 ± 7.3	34.1 ± 8.0	34.2 ± 8.6
Estimated 24-hour salt intake, g/day	8.8 ± 2.1	8.7 ± 2.0	8.7 ± 2.2	8.8 ± 2.0
Estimated 24-hour K intake, mg/day	1695.4 ± 424.1	1685.2 ± 370.5	1731.1 ± 406.8	1738.6 ± 437.8
Characteristics obtained at annual health checkup				
Body mass index, kg/m^2^	22.9 ± 3.9	22.3 ± 3.5	22.5 ± 3.9	22.8 ± 3.7
Systolic blood pressure, mmHg	115.9 ± 14.4	115.4 ± 15.8	115.6 ± 14.7	116.2 ± 14.1
Diastolic blood pressure, mmHg	72.5 ± 12.5	71.6 ± 12.0	73.1 ± 11.4	73.0 ± 10.9
Triglyceride, mg/dL	97.7 ± 54.8	90.1 ± 61.1	101.8 ± 76.2	98.7 ± 59.8
HDL-cholesterol, mg/dL	65.8 ± 16.6	68.3 ± 18.9	64.0 ± 15.0	66.4 ± 19.4
LDL-cholesterol, mg/dL	114.0 ± 28.7	122.2 ± 30.0	121.2 ± 34.0	122.8 ± 30.6
AST, mg/dL	22.8 ± 9.3	23.1 ± 16.2	26.7 ± 42.6	23.4 ± 10.7
ALT, mg/dL	26.0 ± 20.9	22.7 ± 13.2	24.9 ± 24.4	26.1 ± 29.4
γ-GTP, mg/dL	44.7 ± 53.4	31.3 ± 29.8	50.6 ± 206.5	38.7 ± 42.7
Hemoglobin A1c, %	5.4 ± 0.4	5.3 ± 0.4	5.4 ± 0.5	5.3 ± 0.3
Serum Cr, mg/dL	0.75 ± 0.16	0.75 ± 0.16	0.74 ± 0.16	0.77 ± 0.16
eGFR, mL/min/1.73m^2^	87.9 ± 11.8	88.1 ± 10.5	88.6 ± 11.7	86.9 ± 10.9
Hypertension, %	14 (16.7)	12 (14.3)	14 (16.1)	15 (17.2)
Use of antihypertensive drug	4 (4.8)	5 (6.0)	8 (9.2)	6 (6.9)
Diabetes, %	2 (2.4)	1 (1.2)	6 (6.9)^※^	0 (0.0)

Data are expressed as mean ± SD or counts (percentages). Characteristics were compared between each two groups using unpaired *t*-tests or chi-square (χ2) tests. ^※^The characteristics of the participants were similar across all groups, except for the prevalence of diabetes between the self-learning and control groups (*P* = 0.01).

Abbreviations: γ-GTP, gamma-glutamyl transpeptidase; ALT, alanine aminotransferase; AST, aspartate aminotransferase; Cr, creatinine; eGFR, estimated glomerular filtration rate; HDL, high density lipoprotein; K, potassium; LDL, low density lipoprotein; Na, sodium; SD, standard deviation.

The mean (SD) duration of the trial period was 2.9 (0.6) months in the online education group, 2.9 (0.6) months in the messaging group, 2.9 (0.5) months in the self-learning group, and 2.9 (0.5) months in the control group. The numbers of participants in each group who completed urine tests at each study timepoint are shown in Supplementary Table S4.

### Changes in urinary measures during the trial

#### Intention-to-treat analysis.

**Figure 2.**
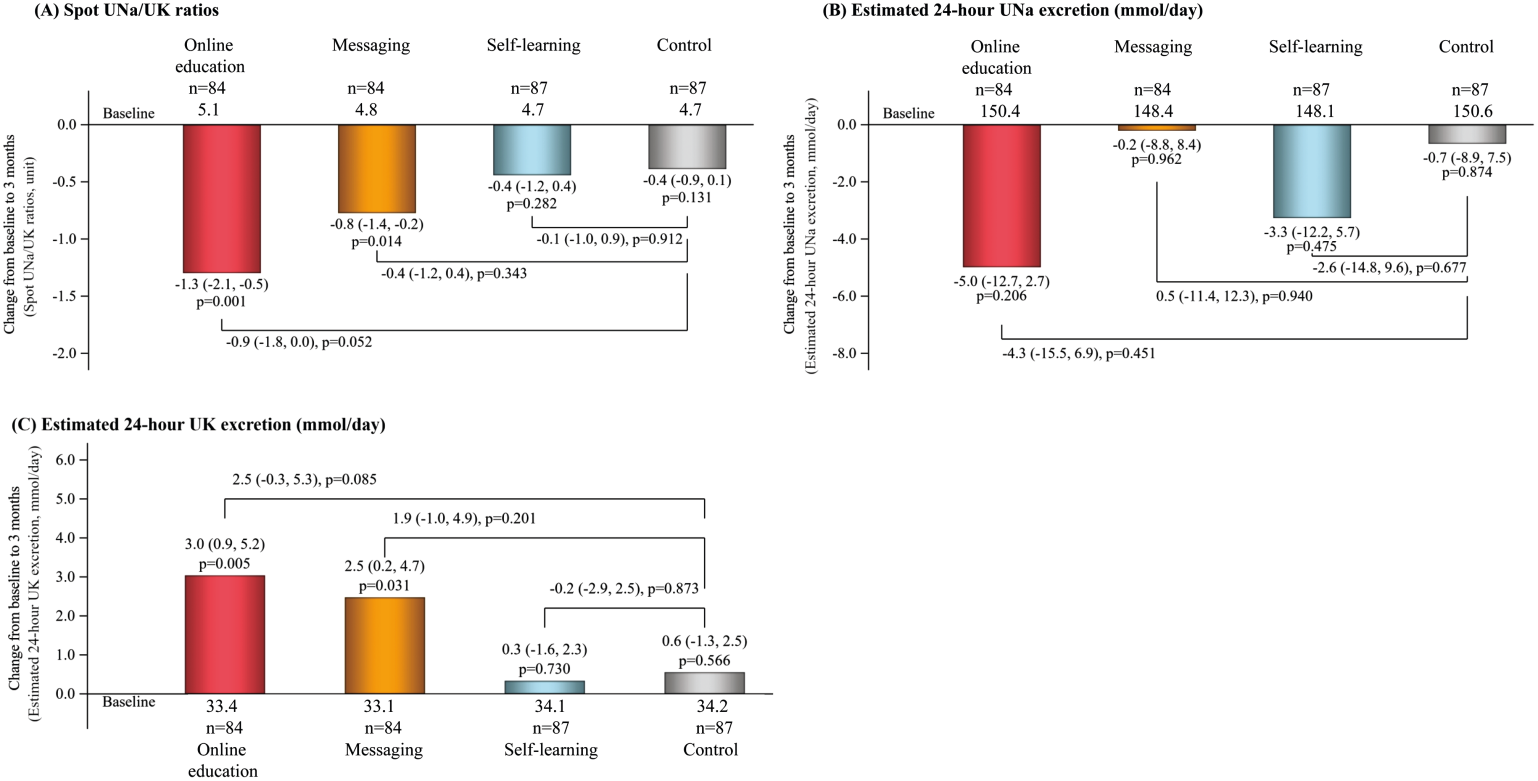
Changes in urinary measures during the trial. Mean changes and the 95% CIs from baseline to Month 3 in spot UNa/UK ratios (**a**), and estimated 24-hour UNa (**b**) and UK excretion (**c**) are shown. To assess the effectiveness of the intervention, mixed models were used to compare the baseline and follow-up data within each group. Changes between baseline and Month 3 in the intervention and control groups were measured using the mean and 95% CIs at baseline and Month 3. For adjustment of multiplicity, hypotheses were tested according to a hierarchical strategy. We imputed missing data for urinary measures at baseline and Month 3 using an iterative Markov chain Monte Carlo method with 20 iterations. UNa, urinary sodium; UK, urinary potassium.

During the 3-month intervention, mean levels of UNa/UK ratios declined by an average of −1.3 (95% CI: −2.1 to −0.5) in the online education group, −0.8 (95% CI: −1.4 to −0.2) in the messaging group, −0.4 (95% CI: −1.2 to 0.4) in the self-learning group, and −0.4 (95% CI: −0.9 to 0.1) in the control group (Figure 2a). The change in spot UNa/UK ratios between the online education and control groups was −0.9 (95% CI: −1.8 to 0.0; *P* = 0.052; Figure 2a). During the intervention, no statistically significant change was observed in estimated 24-hour UNa excretion between the online education and control groups in all groups (Figure 2b). Estimated 24-hour UK excretion increased in the online education group (mean 3.0 mmol/day and 95% CI: 0.9 to 5.2) and in the messaging group (mean 2.5 mmol/day and 95% CI: 0.2 to 4.7) after the intervention. The increase in estimated 24-hour UK excretion was larger in the online education group (mean + 2.5 mmol/day (95% CI: −0.3 to 5.3), *P* = 0.085; Figure 2c). There was no evidence of interaction between each intervention group and sex, age (<40 vs. ≥40 years), body mass index (<25 vs. ≥25 kg/m^2^), baseline UNa/UK ratios and estimated 24-hour UNa and UK excretion above or below median levels, and study location in association with changes in UNa/UK ratios and in estimated 24-hour UNa and UK excretion (all *P* ≥ 0.07). Therefore, we did not conduct stratified analyses or present subgroup-specific results in a supplemental table or figure. The full analysis set yielded similar results, with comparable point estimates for changes in urine metrics between the online education and control groups (Supplementary Figure S4).

### Sensitivity analyses

When we performed analyses without imputing missing urinary measures (Supplementary Figure S5), the change in UNa/UK ratios between the online education and control groups was −0.9 (95% CI: −1.8 to 0.0; *P* = 0.049). Similarly, using urinary measures obtained from the screening exams for missing baseline values (Supplementary Figure S6), the change in UNa/UK ratios was −0.8 (95% CI: −1.7 to 0.1; *P* = 0.085). When adjusting for baseline urinary measures, age, sex, and variables obtained during health checkups (Supplementary Figure S7), the change was −0.6 (95% CI: −1.2 to 0.0; *P* = 0.041). These results are consistent with those from the primary analyses, which demonstrated a significant reduction in UNa/UK ratios in the online education group compared to the control group.

## DISCUSSION

The current study indicates that online counseling leads to a reduction in the spot Una/UK ratio. Participants of the online education group (who received both text messages and online nutritional information) experienced a larger decrease in UNa/UK ratios compared to the control group, although the difference was not statistically significant. The online education group showed an increase in estimated 24-hour UK excretion compared to the control group, although the difference was not statistically significant. In comparison, changes in estimated 24-hour UNa excretion were not statistically significant in any of the groups during the intervention.

Online counseling typically offers a customized experience, adapting to each individual’s unique needs and situation, potentially leading to increased involvement and enhanced comprehension of methods for decreasing sodium intake while increasing potassium consumption.^20,21^ Furthermore, online counseling allows for real-time interaction, which may foster the development of a collaborative and supportive relationship between a participant and counselor more effectively compared to messaging and self-learning through written information sources. However, our study revealed that combining self-monitoring of sodium and potassium intake with ICT-based strategies, including online nutritional education, failed to produce a statistically significant change in spot UNa/UK ratios in healthy participants. Several factors may explain this outcome. First, the observed effect size—a 0.9 reduction in the UNa/UK ratio—was smaller than the anticipated 1.0, suggesting the study may have been underpowered to detect meaningful changes. Second, the baseline estimated 24-hour urinary sodium excretion in our study population appeared lower than that reported in some other Asian populations.^8,22^ This may have contributed to the lack of a significant reduction in urinary sodium excretion, as participants may have had less room for further reduction in sodium intake. Third, intraindividual variation in spot UNa/UK ratios, which may have been affected by potential inconsistencies in urine sample collection conditions that were not verified for uniformity across participants, could have influenced the observed intervention effects. Moreover, using a single spot urine measurement at baseline and follow-up may have limited precision, as biological variation and daily fluctuations in sodium and potassium excretion could introduce variability. Fourth, while participants were initially screened based on UNa/UK ratios >4.0, a second urine test conducted before the intervention was used as the baseline measurement. As a result, some participants exhibited lower UNa/UK ratios at baseline than during screening. This natural variability in sodium and potassium excretion may have attenuated the observed intervention effects. Finally, differences in the interpretation of spot UNa and UK estimates at the population versus individual level may have influenced the findings. While spot UNa/UK ratios are useful for monitoring population-level trends, they may be subject to variability when applied to individual assessments due to factors such as hydration status, timing of sample collection, and other individual-specific variables.

In this study, the estimated 24-hour UK excretion significantly increased in the online education and messaging groups, while UNa excretion remained unchanged. This suggests that participants may have consumed more potassium-rich foods, but sodium reduction remains challenging due to its prevalence in the food supply. In Japanese cuisine, higher potassium intake often correlates with increased sodium use in seasonings (e.g., soy sauce and miso), making salt reduction difficult.^23,24^ Future research should explore strategies to boost vegetable intake while minimizing sodium, including low-sodium seasoning alternatives and personalized nutrition approaches considering taste preferences, genetics, and lifestyle factors.

The feasibility and effectiveness of the three intervention strategies warrant consideration. Online education provides personalized interaction and structured feedback, which may enhance engagement and comprehension. Messaging interventions, while offering frequent reinforcement, may have been less effective in promoting sustained behavior change. Self-learning through educational leaflets, although easy to implement, provided the least engagement and lacked ongoing support, which may explain the smaller observed effects. While all strategies were feasible, their effectiveness may vary based on individual motivation, digital literacy, and personal preferences. Future research should assess the long-term impact and scalability of these approaches in different populations.

This trial’s strengths include a high follow-up rate (>95%) and comparable study groups. However, findings may not generalize to those without high sodium and low potassium intake, older adults (>65 years), or individuals with comorbidities. The 3-month duration limits the assessment of long-term effects, and unmeasured factors (e.g., socioeconomic status, physical activity, and sleep) may have influenced dietary behavior.^2,23^ Although BP data were available from prior health checkups, they were collected before enrollment, preventing assessment of BP changes’ impact on UNa/UK ratios. Self-regulation is also a potential confounder. A person who has high intrinsic motivation for improving their health or preventing future disease may show improved outcomes after being given test results and a leaflet, while someone with low intrinsic motivation may benefit from individualized health coaching (or might not adhere to a more time-intensive intervention).

In this trial involving healthy persons, we provide evidence that the effectiveness of an intervention to reduce sodium intake and increase potassium intake by promoting self-learning may improve when additional ICT-based methods are utilized and regular testing is undertaken. The inclusion of periodic text messaging and online nutritional education to supplement written educational information and test results may help individuals be more mindful of and accountable regarding their dietary choices and how they can affect their personal risk for developing hypertension.

## Supplementary material

Supplementary materials are available at *American Journal of Hypertension* (http://ajh.oxfordjournals.org).

hpaf049_suppl_Supplementary_Tables_S1-S4_Figures_S1-S7

## Data Availability

The datasets used and/or analyzed during the current study are not an open access.
