# Supplementary material for: A Randomized Clinical Trial of ICT-based Interventions for Sodium and Potassium Regulation in Healthy Adults
Source: Am J Hypertens. 2025 Apr 11;38(8):588–94. doi: 10.1093/ajh/hpaf049 (PMC12260158; doi:10.1093/ajh/hpaf049)
Supplement: hpaf049_suppl_Supplementary_Tables_S1-S4_Figures_S1-S7 [file hpaf049_suppl_supplementary_tables_s1-s4_figures_s1-s7.docx]

**Supplemental Material**

**Intervention**

Participant groups and interventional variables are described below.

**Online education group.** Personalized interactive communication to reduce dietary sodium and increase potassium intake was provided by registered dieticians via online or videoconference using MedicallyApp (MEDCARE, INC) at four timepoints: baseline, 1 and 2 months after baseline, and at the end of the intervention. Participants underwent spot urine testing at 1 and 2 months after the baseline, and feedback and communication of test results was provided by registered dieticians. Participants were also sent online text messages twice a week (**Table S1**) and provided with a booklet containing specific information regarding how to reduce dietary salt and increase potassium intake. Participants were asked to set specific goals to reduce dietary sodium and increase potassium intake (**Table S2**). Incentives were provided only to participants who self-reported that they felt like they achieved goals (**Table S3**) to motivate consumption of foods low in sodium and high in potassium.

**Messaging group.** Participants randomized to this group underwent spot urine testing at 1 and 2 months after baseline, and test results were returned to them. Based on the results, participants were asked to set specific goals to reduce dietary sodium and increase potassium intake (**Table S2**). Participants who felt that they achieved their goals were provided incentives (**Table S3**). Participants also received online text messages twice a week to encourage them to consume foods low in sodium and high in potassium (**Table S1**).

**Self-learning group.** Participants randomized to this group were provided with a leaflet that contained specific information regarding how to reduce dietary salt and increase potassium intake as recommended by the Japanese Society of Hypertension.^1^ Results of spot urine tests conducted at baseline were returned to participants, and participants were asked to change their eating habits based on the guidance provided in the leaflet. Participants in this group underwent no additional spot urine testing and did not receive online education or text messages related to the study during the intervention.

**The control group.** Participants in this group did not receive results of spot urine testing at baseline, and thus were not aware whether their diets had been confirmed to be high in sodium and/or low in potassium. During the intervention, control group members did not receive any dietary instructions or restrictions, and did not receive additional spot urine tests, online education, study-related text messages, the booklet, or leaflet.

**Estimation of 24-hour sodium and potassium intake**

During both the baseline assessment and resurvey visit conducted 3 months after baseline, initial morning spot urine samples were collected from all participants (see **Figure S1** for urine collection procedures). Samples were transferred from the collection vessels to the coordinating center laboratory (Healthcare Systems, Inc) on the day of sampling. Urinary analyses were performed within 72 hours after sample collection using a TBA120FR (CANON MEDICAL SYSTEMS CORPORATION). The concentration of urinary sodium and potassium was measured using the electrode method, and urinary creatinine (Cre) using an enzymatic method. Estimated 24-hour UNa excretion (mmol/day) was calculated from spot UNa and urinary Cre values using the following formula: 21.98 × {Na (mEq/l) × estimated 24-hours Cre excretion / [Cre (mg/dl) × 10]} ^0.392^ , where 24-hour Cre excretion was predicted using the following formula: body weight (kg) × 14.89 + body height (cm) × 16.14 − age × 2.04 − 2244.45.^2^ Estimated 24-hour UK excretion (mmol/day) was calculated from spot UK and urinary Cre values using the following formula: 7.59 × {K (mEq/l) × estimated 24-hours Cre excretion / [Cre (mg/dl) × 10]^0.431^ Estimated 24-hour salt intake (g/day) was calculated as: estimated 24-hour UNa excretion × 0.0585. Estimated 24-hour K intake (mg/day) was calculated as: estimated 24-hour UK excretion × 39.1/0.77.^3^

**Other measurements**

In 2008, the Japanese government initiated a health care strategy that targeted early diagnosis and intervention for metabolic syndrome (Specific Health Checkups and Guidance System [Tokutei-Kensin]).^4^ Employers in Japan have a legal obligation to offer annual health screenings to their employees. At annual health checkups, several measurements were collected using standardized protocols across trial centers, including history of hypertension and diabetes, body mass index, BP, fasting laboratory values, and urinalysis by the dipstick method. Estimated glomerular filtration rate (eGFR) was derived using the Chronic Kidney Disease Epidemiology Collaboration (CKD-EPI) equation modified by a Japanese coefficient.^5^ Diabetes mellitus was defined as fasting plasma glucose ≥126 mg/dL, random plasma glucose ≥200 mg/dL, hemoglobin A1c ≥6.5%, or self-reported use of diabetes medications. Hypertension was defined as Systolic BP (SBP) ≥140 mm Hg, Diastolic BP (DBP) ≥90 mm Hg, or taking antihypertensive medication. Since all participants underwent health checkups before being screened for eligibility to participate in the current study (i.e., health checkup data were obtained at a median and quartile of 142 and 65-266 days before participants were recruited for the current RCT), we used the health checkup data to provide participant characteristics.

**Statistical analysis**

The individual responsible for statistical analysis (H.K) was initially blinded to the allocation details. In accordance with the prespecified statistical analysis plan, three hypotheses for the primary and secondary end points were tested hierarchically. At the forefront of this hierarchy was the online education group, considered the most critical component of our intervention. However, achieving persuasive outcomes in both the messaging group and the self-learning group would also significantly contribute to our comprehensive understanding of the distinct value each component brings to the overall treatment strategy. This tiered approach allowed us to assess the individual and collective impacts of the various intervention components, with a particular emphasis on the primacy of online education while still recognizing the potential additive benefits of messaging and self-learning in supporting healthy dietary changes.

**References**

1. Umemura S, Arima H, Arima S, Asayama K, Dohi Y, Hirooka Y, Horio T, Hoshide S, Ikeda S, Ishimitsu T, Ito M, Ito S, Iwashima Y, Kai H, Kamide K, Kanno Y, Kashihara N, Kawano Y, Kikuchi T, Kitamura K, Kitazono T, Kohara K, Kudo M, Kumagai H, Matsumura K, Matsuura H, Miura K, Mukoyama M, Nakamura S, Ohkubo T, Ohya Y, Okura T, Rakugi H, Saitoh S, Shibata H, Shimosawa T, Suzuki H, Takahashi S, Tamura K, Tomiyama H, Tsuchihashi T, Ueda S, Uehara Y, Urata H, Hirawa N. The Japanese Society of Hypertension Guidelines for the Management of Hypertension (JSH 2019). *Hypertens Res* 2019; 42(9)**:** 1235-1481.

2. Tanaka T, Okamura T, Miura K, Kadowaki T, Ueshima H, Nakagawa H, Hashimoto T. A simple method to estimate populational 24-h urinary sodium and potassium excretion using a casual urine specimen. *J Hum Hypertens* 2002; 16(2)**:** 97-103.

3. Tasevska N, Runswick SA, Bingham SA. Urinary potassium is as reliable as urinary nitrogen for use as a recovery biomarker in dietary studies of free living individuals. *J Nutr* 2006; 136(5)**:** 1334-1340.

4. Matsuda S. [Health check up and health care advice with a particular focus on the metabolic syndrome--background and overview]. *Nihon Rinsho* 2008; 66(7)**:** 1405-1412.

5. Horio M, Imai E, Yasuda Y, Watanabe T, Matsuo S. Modification of the CKD epidemiology collaboration (CKD-EPI) equation for Japanese: accuracy and use for population estimates. *Am J Kidney Dis* 2010; 56(1)**:** 32-38.

| **Table S1. Online text message topics and links provided twice a week to participants in the online education and messaging groups** | | | | |
| --- | --- | --- | --- | --- |
| The first month | The first week | 1st day | Introduction to increasing potassium intake; how we can eat 350g or more of vegetables per day. | <https://contents.jsh-natokari.jp/contents/contents4/increase-potassium-veg/q> |
|  |  |  | Introduction to increasing potassium intake; how to change one’s diet to include one more serving of vegetables per day. | <https://contents.jsh-natokari.jp/contents/contents4/increase-potassium-veg/> |
|  |  | 2nd day | Introduction on cooking techniques to retain potassium in foods: When preparing vegetables, it is better to use a microwave oven rather than boiling to prevent potassium dissolving in water. | <https://contents.jsh-natokari.jp/contents/contents4/increase-potassium-veg/b/> |
|  |  |  | Additional cooking techniques to retain potassium in foods: When boiling vegetables and potatoes, use as little water as possible, and use the cooking liquid as a soup stock instead of discarding it. | https://contents.jsh-natokari.jp/contents/contents4/increase-potassium-veg/c/ |
|  | The second week | 1st day | Avoiding salty soups with noodles; the approximate salt content in noodle soups, and how to prepare versions lower in salt. | <https://contents.jsh-natokari.jp/contents/contents3/leave-noodle-soup/> |
|  |  |  | Avoiding Japanese pickles; the approximate salt content in Japanese pickles. | <https://contents.jsh-natokari.jp/contents/contents3/refrain-pickles/> |
|  |  | 2nd day | Limiting daily consumption of miso soup to one cup or less; miso paste is high in salt; how to cook miso soup using less salt. | <https://contents.jsh-natokari.jp/contents/contents3/miso-soup-1-cup-a-day/> |
|  |  |  | Adjusting seasoning to taste; Introduction how to adjust seasoning. | <https://contents.jsh-natokari.jp/contents/contents3/stop-use-seasonings-without-tasting/> |
|  | The third week | 1st day | Avoiding seasonings that are high in salt; overview of the amount of salt in prepared seasonings. | <https://contents.jsh-natokari.jp/contents/contents3/use-low-salt-type-seasonings/> |
|  |  |  | Avoiding processed foods, e.g., fish paste, ham and sausage, and ready to eat side dishes with high salt; overview of the amount of salt content in various foods. | <https://contents.jsh-natokari.jp/contents/contents3/refrain-processed-foods/> |
|  |  | 2nd day | Increasing potassium intake by eating fruits; comparing the amount of potassium found in fresh fruits, canned fruits, and juices. | <https://contents.jsh-natokari.jp/contents/contents4/increase-potassium-fruits/a/> |
|  |  |  | Increasing potassium intake by eating dried fruits; comparing the amount of potassium in dried fruits. | <https://contents.jsh-natokari.jp/contents/contents4/increase-potassium-fruits/b/> |
|  |  | Additionary | How much fruit per day? Fruits are higher in carbohydrates and calories than vegetables, so be careful not to eat too much; recommended daily intake of fruit. | <https://contents.jsh-natokari.jp/contents/contents4/increase-potassium-fruits/j/> |
|  | The fourth week | 1st day | Using vinegar and spices instead of salt to add flavor; types of spices and how to cook with them. | <https://contents.jsh-natokari.jp/contents/contents3/use-vinegar-and-spices/> |
|  |  |  | Increasing consumption of aromatic vegetables; types of aromatic vegetables and how to cook them. | <https://contents.jsh-natokari.jp/contents/contents3/use-aroma-veg/> |
|  |  | 2nd day | Increasing potassium intake by eating legumes; approximate potassium content in legumes, and how to cook them. | <https://contents.jsh-natokari.jp/contents/contents4/increase-potassium-valanced-diet/b/> |
|  |  |  | Noodles and rice bowl dishes tend to be high in salt and low in potassium. Try to combine noodles and rice bowl dishes with side dishes such as salad or small bowls of vegetables. | <https://contents.jsh-natokari.jp/contents/contents4/increase-potassium-valanced-diet/a/> |
| The second month | The first week | 1st day | The sodium/potassium (Na/K) ratio and why it is important. Excess sodium intake and insufficient potassium intake are associated with high blood pressure, heart diseases, and strokes. | <https://contents.jsh-natokari.jp/contents/contents1/about-natokari/> |
|  |  |  | The urine sodium/potassium (Na/K) ratio is the ratio of sodium (Na) to potassium (K) excreted in urine. It can be used to estimate dietary salt and potassium intake through urinalysis. | <https://contents.jsh-natokari.jp/contents/contents1/about-natokari-ratio/> |
|  |  | 2nd day | What is urinary sodium excretion? Since most of the sodium we consume is excreted in urine, dietary salt intake can be estimated by measuring urinary sodium excretion. | <https://contents.jsh-natokari.jp/contents/contents1/about-urine-sodium/> |
|  |  |  | What is urinary potassium excretion? Since about 70% of our potassium intake is excreted in urine, dietary potassium intake can be estimated by measuring urinary potassium excretion. | <https://contents.jsh-natokari.jp/contents/contents1/about-urine-potassium/> |
|  | The second week | 1st day | Health article—Dietary salt intake and blood pressure in a representative Japanese population: baseline analyses of NIPPON DATA80. Miura K, et al. J Epidemiol. 20 Suppl 3: S524-30. 2010​ | <https://contents.jsh-natokari.jp/contents/contents2/relation-sodium-blood-pressure/> |
|  |  |  | Health article—Long-term risk of BP values above normal for cardiovascular mortality: a 24-year observation of Japanese aged 30 to 92 years. Takashima N, et al. J Hypertens. 30(12):2299-306. 2012 | <https://contents.jsh-natokari.jp/contents/contents2/high-blood-pressure/> |
|  |  | 2nd day | Health article—Sodium/potassium ratio was associated with blood pressure: possibility of population approach for sodium/potassium ratio reduction in health checkup. Kogure M, et al. Hypertens Res. 44: 225–231. 2020 | <https://contents.jsh-natokari.jp/contents/contents2/natkari-ratio-and-high-blood-pressure/> |
|  |  |  | Health article—Dietary sodium-to-potassium ratio as a risk factor for stroke, cardiovascular disease and all-cause mortality in Japan: the NIPPON DATA80 cohort study. Okayama A, et al. BMJ Open. 2016​ | <https://contents.jsh-natokari.jp/contents/contents2/stroke-and-natokari-ratio/> |
|  | The third week | 1st day | Health article—A clinical trial of the effects of dietary patterns on blood pressure. DASH Collaborative Research Group. Apple L. J. et al, N Engl J Med. 17;336(16):1117-24. 1997 | <https://contents.jsh-natokari.jp/contents/contents2/veg-fruits-and-blood-pressure/> |
|  |  |  | Health article—NIPPON DATA80 Research Group. Fruit and vegetable intake and mortality from cardiovascular disease in Japan: a 24-year follow-up of the NIPPON DATA80 Study. Okuda N, et al. Eur J Clin Nutr. 69: 482-8. 2015 | <https://contents.jsh-natokari.jp/contents/contents2/veg-fruirs-and-circulatory-disease/> |
|  |  | 2nd day | The Ministry of Health, Labour, and Welfare in Japan’s recommendation for daily intake of vegetables is 350g or more. | <https://contents.jsh-natokari.jp/contents/contents4/increase-potassium-veg/q/> |
|  |  |  | Visualization of 350g of vegetables. | https://contents.jsh-natokari.jp/contents/contents4/increase-potassium-veg/r |
|  | The fourth week | 1st day | Overview—the average vegetable intake of adults in Japan. | <https://contents.jsh-natokari.jp/contents/contents4/increase-potassium-veg/x/> |
|  |  |  | Reasons for consuming 350g or more of vegetables per day—we need to consume an adequate amount of potassium, dietary fiber, and vitamins to maintain a healthy body, including preventing cardiovascular diseases and cancer. | <https://contents.jsh-natokari.jp/contents/contents4/increase-potassium-veg/u/> |
|  |  | 2nd day | The main reasons for inadequate vegetable intake—missed meals and eating out frequently. | <https://contents.jsh-natokari.jp/contents/contents4/increase-potassium-veg/y/> |
|  |  |  | Focusing on "one more serving" of vegetables; how to increase consumption. | <https://contents.jsh-natokari.jp/contents/contents4/increase-potassium-veg/v/> |
| The third month | The first week | 1st day | Low-salt seasonings we can buy at supermarkets. | <https://contents.jsh-natokari.jp/contents/contents7/use-low-salt-food/a/> |
|  |  |  | Low-salt soup and noodles we can buy at supermarkets. | <https://contents.jsh-natokari.jp/contents/contents7/use-low-salt-food/d/> |
|  |  | 2nd day | Increase potassium intake by choosing brown rice or multigrain rice rather than white rice. | <https://contents.jsh-natokari.jp/contents/contents4/increase-potassium-valanced-diet/e/> |
|  |  |  | Increase potassium intake by mixing juice with milk and fruits. | <https://contents.jsh-natokari.jp/contents/contents4/increase-potassium-fruits/h/> |
|  | The second week | 1st day | Reduce dietary salt intake by eating more tomatoes. | <https://contents.jsh-natokari.jp/contents/contents3/low-salt-cooking/a/> |
|  |  |  | Reduce dietary salt intake by eating more onions. | <https://contents.jsh-natokari.jp/contents/contents3/low-salt-cooking/c/> |
|  |  | 2nd day | Reduce dietary salt and increase potassium intake; the 'New-Washoku' and recipes. | <https://contents.jsh-natokari.jp/contents/contents3/reduce-salt-with-milk/g/> |
|  |  |  | Reduce dietary salt and increase potassium intake; How to cook using dairy milk products in Washoku. | https://contents.jsh-natokari.jp/contents/contents3/reduce-salt-with-milk/e/ |
|  | The third week | 1st day | Increasing potassium intake; good vegetable juice choices. | <https://contents.jsh-natokari.jp/contents/contents4/increase-potassium-drink/g/> |
|  |  |  | Increasing potassium intake; potassium content in vegetable juices. | <https://contents.jsh-natokari.jp/contents/contents4/increase-potassium-drink/j/> |
|  |  | 2nd day | Increasing potassium intake; potassium content in coffee. | <https://contents.jsh-natokari.jp/contents/contents4/increase-potassium-drink/c/> |
|  |  |  | Coffee is also rich in polyphenols. | <https://contents.jsh-natokari.jp/contents/contents4/increase-potassium-drink/f/> |
|  | The fourth week | 1st day | Three types of dishes can help people maintain an ideal nutritional balance: a staple dish, such as rice, is the major source of energy; a main dish, such as meat, fish, etc., supplies protein and fat; and a side dish mainly consists of vegetables/mushrooms and is a source of vitamins/minerals. | <https://contents.jsh-natokari.jp/contents/contents4/increase-potassium-valanced-diet/g/> |
|  |  |  | The concept of "staple dish/main dish/side dish" in Japan. | <https://contents.jsh-natokari.jp/contents/contents4/increase-potassium-valanced-diet/f/> |
|  |  | 2nd day | Reducing dietary salt intake; salt content in various foods. | <https://contents.jsh-natokari.jp/contents/contents3/beware-of-salty-foods/> |
|  |  |  | Look at the Label! A label lists elements in the order of energy, protein, fat, carbohydrates, and sodium, provided that sodium is indicated as 'salt equivalent.' | <https://contents.jsh-natokari.jp/contents/contents3/check-salt-equivalent/> |

| **Table S2. Specific goals to reduce dietary sodium and increase potassium intake** | |
| --- | --- |
| 1 | Eat one serving of fruit at least once every two days. |
| 2 | Eat one or two serving of fruits every day. |
| 3 | Eat the entire portion of a vegetable side dish. |
| 4 | Eat a side dish of vegetables every day. |
| 5 | Eat a side dish of vegetables at every meal. |
| 6 | Eat a meal that includes staple, main and side dishes at least twice a day. |
| 7 | Eat at least 5 servings (350g) of vegetables every day. |
| 8 | Drink more than three cups of potassium-rich beverages such as Japanese tea, coffee, and black tea every day (sugarless). |
| 9 | Drink one glass of milk (200cc) every day. |
| 10 | Drink more than one glass of salt-free vegetable juice (200cc) every day. |
| 11 | Avoid salty noodle soups. |
| 12 | Avoid Japanese pickles. |
| 13 | Eat no more than one cup of miso soup per day. |
| 14 | Adjust seasonings to taste. |
| 15 | Reduce use of salty seasonings. |
| 16 | Reduce use of soy sauce. |
| 17 | Avoid processed and ready-to-eat side dishes that are high in salt. |
| 18 | Do not have salty seasonings at the table during mealtimes. |
| 19 | Use vinegar and spices more often to add flavor to dishes. |
| 20 | Eat aromatic vegetables often. |
| 21 | Go to restaurants certified for Healthy Meal and Food Environment “Smart meal” when eating out or for home meal replacement (see notebook). |
| 22 | Check Nutrition Facts labels when purchasing food or eating out. |
| 23 | Use low salt products listed by the Japan Society of Hypertension (see notebook). |

| **Table S3. Incentives provided to participants who achieved their goals** | |
| --- | --- |
| Ajinomoto Low Salt Hondashi | 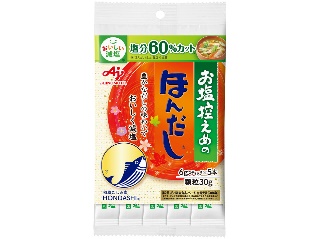 |
| Ajinomoto Whole Chicken Stock Low Salt | 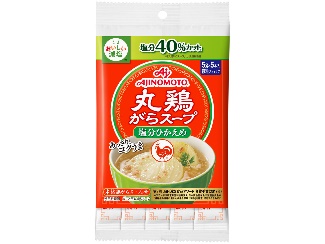 |

| **Table S4. Urinary measures collected at each study timepoint** | | | | | | | |
| --- | --- | --- | --- | --- | --- | --- | --- |
|  | **Screening** | **Baseline (month 0)** | **Number of imputed**  **observations for urine measures at baseline** | **Month 1** | **Month 2** | **Month 3** | **Number of imputed**  **observations for urine measures at month 3** |
| Online education | 84 | 84  (100%) | 0  (0%) | 77  (91.7%) | 74  (88.1%) | 80  (95.2%) | 4  (4.8%) |
| Messaging | 84 | 83  (98.8%) | 1  (1.2%) | 78  (92.6%) | 75  (89.3%) | 80  (95.2) | 4  (4.8%) |
| Self-learning | 87 | 87  (100%) | 0  (0%) | Not Applicable | Not Applicable | 85  (97.7%) | 2  (2.3%) |
| Control | 87 | 86  (98.9%) | 1  (1.1%) | Not Applicable | Not Applicable | 85  (97.7) | 2  (2.3%) |

**Figure S1**

**Figure S1. Urine collection procedures.**

To ensure consistency in urine sample collection, all participants were provided with detailed instructions (Figure S1) on how to properly collect their samples. These standardized instructions were distributed to all participants to maintain uniformity in sample collection procedures. First-morning urine samples were collected both at baseline and during the final survey. The same instructions and conditions were applied for all subsequent measurements to ensure consistency across all time points.

**Figure S2**

**
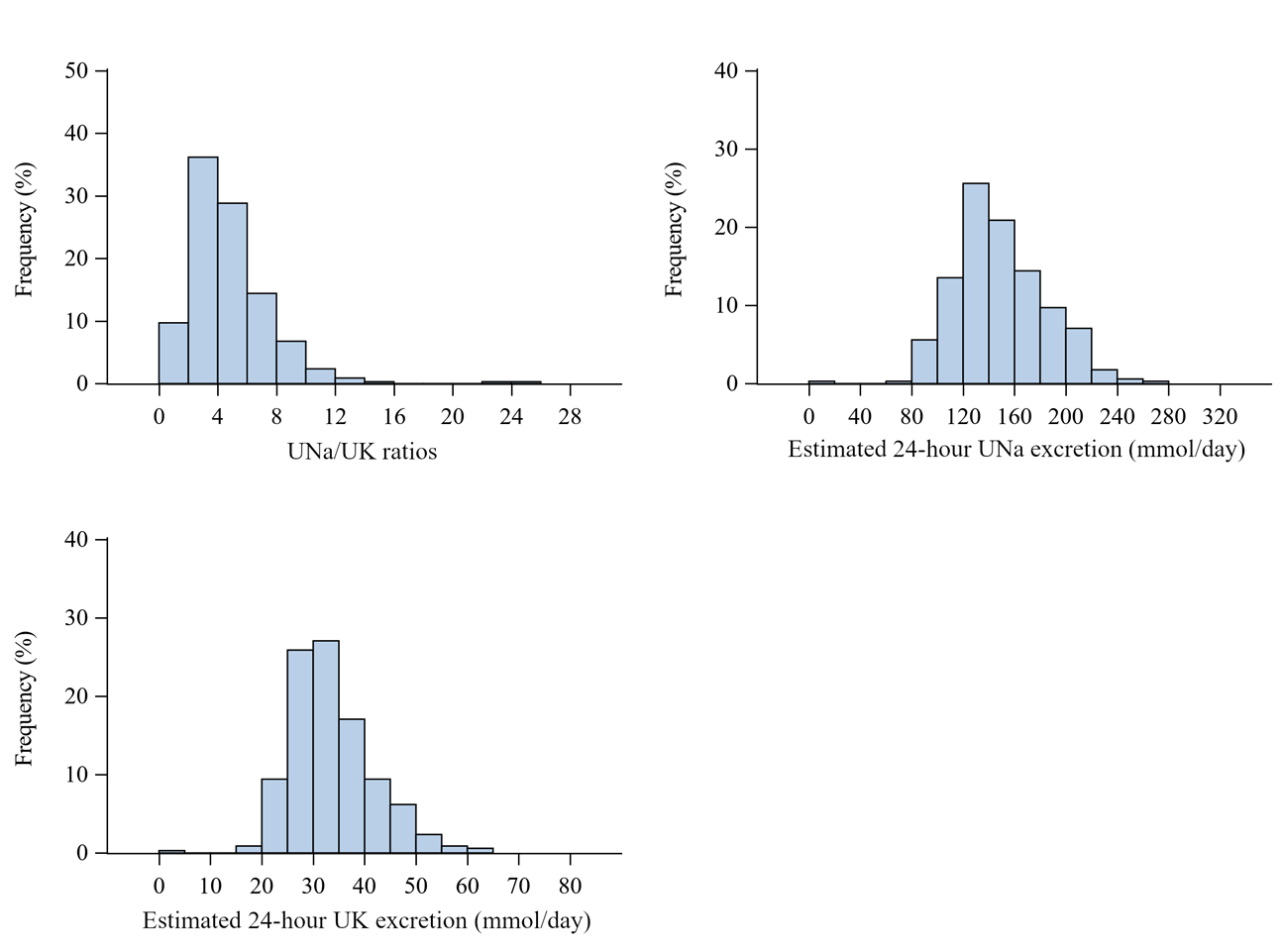
**

**Figure S2. The distribution of spot UNa/UK ratios, and estimated 24-hour UNa and UK excretion at baseline**

The distributions of spot UNa/UK ratios and estimated 24-hour UNa and UK excretion are shown. Estimated 24-hour UNa excretion (mmol/day) was calculated from spot UNa and urinary Cr values using the following formula: 21.98 × {Na (mEq/l) × estimated 24-hour Cr excretion / [Cr (mg/dl) × 10]}^0.392^ , where the 24-hour Cr excretion was predicted using the following formula: body weight (kg) × 14.89 + body height (cm) × 16.14 − age × 2.04 − 2244.45 (Tanaka T, et al. J Hum Hypertens. 2002;16:97–103). Estimated 24-hour UK excretion (mmol/day) was calculated from spot UK and urinary Cr values using the following formula: 7.59 × {K (mEq/l) × estimated 24-hour Cr excretion / [Cr (mg/dl) × 10]}^0.431^.

UNa=Urinary sodium; UK=Urinary potassium; Cr=creatinine

**Figure S3**


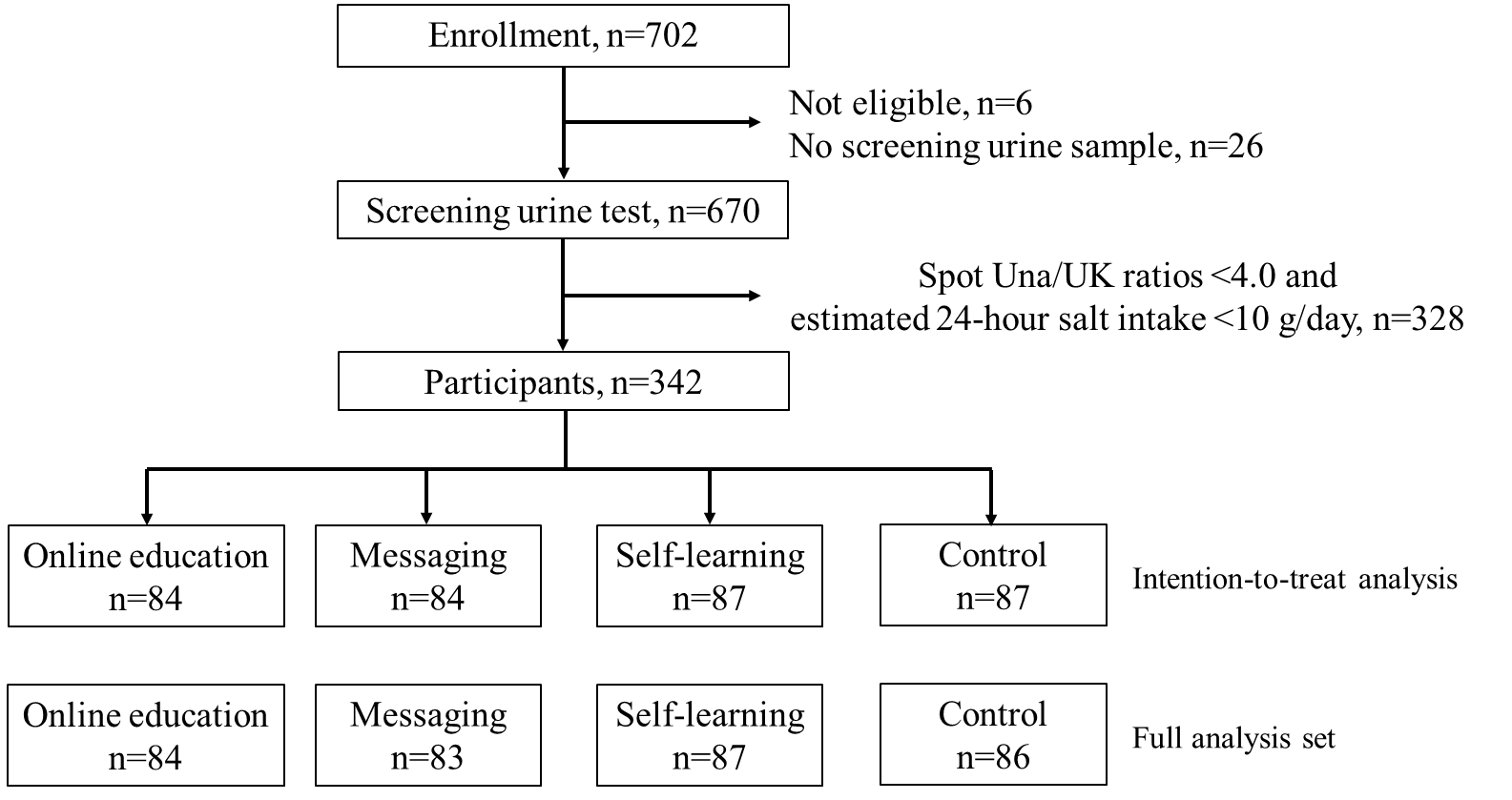


**Figure S3. Sample derivation for the current study.**

In the full analysis set, one individual from the messaging group and another from the control group had missing baseline urine tests. We excluded these two participants and repeated the analyses.

UNa=Urinary sodium; UK=Urinary potassium.

**Figure S4**

**Figure S4. Changes in urine measures during the trial (full analysis set)**

We excluded two participants (one from the messaging group and one from the control group) due to the absence of baseline urine tests. Mean changes and the 95% CIs from baseline to month 3 in spot UNa/UK (Panel A), and estimated 24-hours UNa (Panel B) and UK excretion (Panel C) are shown. To assess the effectiveness of the intervention, mixed models were used to compare the baseline and follow-up data within each group. Changes between the baseline and month 3 in the intervention and control groups were measured with the mean and 95% CIs at baseline and month 3. For adjustment of multiplicity, the hypotheses were tested according to a hierarchical strategy. We imputed missing data for urinary measures at month 3, using an iterative Markov chain Monte Carlo method with 20 iterations.

UNa=Urinary sodium; UK=Urinary potassium.

**Figure S5**

**Figure S5. Changes in urinary measures during the trial (without imputing for missing measures)**

We performed analyses without imputing missing urinary measures.  Mean changes and the 95% CIs from baseline to Month 3 in spot UNa/UK ratios (Panel A), and estimated 24-hour UNa (Panel B) and UK excretion (Panel C) are shown. To assess the effectiveness of the intervention, mixed models were used to compare the baseline and follow-up data within each group. Changes between baseline and Month 3 in the intervention and control groups were measured using the mean and 95% CIs at baseline and Month 3. For adjustment of multiplicity, hypotheses were tested according to a hierarchical strategy.

UNa=Urinary sodium; UK=Urinary potassium.

**Figure S6**

**Figure S6. Changes in urinary measures during the trial (using urinary measures at screening for missing baseline measures)**

We used urinary measures obtained from the screening tests for missing urinary measures at baseline. Mean changes and 95% CIs from baseline to Month 3 in spot UNa/UK ratios (Panel A), and estimated 24-hour UNa (Panel B) and UK excretion (Panel C) are shown. To assess the effectiveness of the intervention, mixed models were used to compare the baseline and follow-up data within each group. Changes between baseline and Month 3 in the intervention and control groups were measured using the mean and 95% CIs at baseline and Month 3. For adjustment of multiplicity, hypotheses were tested according to a hierarchical strategy.

UNa=Urinary sodium; UK=Urinary potassium.

**Figure S7**

**Figure S7. Changes in urinary measures during the trial (adjustments for covariates)**

We adjusted for the baseline urinary data, age, sex, and variables obtained in the health checkups (i.e., body mass index, eGFR, hypertension, and diabetes). Mean changes and 95% CIs from baseline to Month 3 in spot UNa/UK (Panel A), and estimated 24-hour UNa (Panel B) and UK excretion (Panel C) are shown. To assess the effectiveness of the intervention, mixed models were used to compare the baseline and follow-up data within each group. Changes between baseline and Month 3 in the intervention and control groups were measured using the mean and 95% CIs at baseline and Month 3. For adjustment of multiplicity, hypotheses were tested according to a hierarchical strategy.

UNa=Urinary sodium; UK=Urinary potassium.
